# Case Report: Filum terminale extradural arachnoid diverticulum associated with tethered cord syndrome in a French Bulldog

**DOI:** 10.3389/fvets.2026.1792192

**Published:** 2026-04-10

**Authors:** Anna Davis, Isabel Marcos Carpio, Kerstin Baiker, Caroline Fina, Patricia Álvarez, Juan J. Mínguez, Christoforos Posporis

**Affiliations:** 1 Neurology and Neurosurgery Department, Independent Vetcare (IVC) Evidensia, Pride Veterinary Referrals, Derby, United Kingdom; 2 Surgery Department, Independent Vetcare (IVC) Evidensia, Pride Veterinary Referrals, Derby, United Kingdom; 3 Histology Department, Veterinary Pathology Group, Bristol, United Kingdom; 4 Diagnostic Imaging Department, Independent Vetcare (IVC) Evidensia, Pride Veterinary Referrals, Derby, United Kingdom

**Keywords:** dynamic MRI, lumbosacral, meningeal cyst, neuropathic pain, spinal cord

## Abstract

Extradural arachnoid diverticula and tethered cord syndrome are rare disorders in dogs, and their simultaneous occurrence with a possible mechanistic association has not been previously reported. A 2-year-old male intact French Bulldog was evaluated for chronic lumbar pain, presumed secondary aggression, and subtle pelvic limb neurological deficits. Magnetic resonance imaging (MRI) revealed a well-defined, extradural, CSF-isointense, cyst-like lesion located at the origin of the *filum terminale externum* and dural sac termination. An additional intradural, elongated fluid-filled lesion positioned within the subarachnoid space, dorsal to the conus medullaris termination in the region of the filum terminale internum was also identified. Dynamic lumbosacral MRI in neutral, flexed and extended positions demonstrated a fixed position of the conus medullaris and dural sac terminations, raising suspicion for associated tethered cord syndrome. Mild dorsal sacral laminar telescoping with ventral bulging of the interarcuate ligament and dynamic vertebral canal stenosis was also noted, without cauda equina compression. Surgical treatment included a left dorsolateral hemilaminectomy at L5–L6 for en bloc removal of the extradural lesion, excision of a dural–vertebral fibrous adhesion identified intra-operatively, and durotomy with transection of the *filum terminale internum*, which did not appear abnormal intra-operatively. A dorsal laminectomy at S1 was also performed to resect the interarcuate ligament. Histopathology revealed a diverticulum composed of dural and arachnoid elements and confirmed the identity of the *filum terminale internum*. A final diagnosis of a type Ib extradural arachnoid diverticulum associated with tethered cord syndrome was made, although concurrent dynamic lumbosacral stenosis due to sacral laminar telescoping could not be excluded. The dog achieved full clinical recovery, with resolution of pain and behavioral abnormalities, and remained normal at 1-year follow-up. This case documents a previously unreported extradural arachnoid diverticulum arising from a pedicle of the meninges of the *filum terminale externum* and dural sac termination. It highlights the potential role of dynamic MRI in assessing conus medullaris mobility in cases with *filum terminale* pathology and shows a favorable outcome following surgical intervention.

## Introduction

Spinal meningeal cysts (SMCs) and tethered cord syndrome (TCS) are rare spinal disorders in dogs and have not previously been reported to occur in association (1–4). Whilst commonly referred to as “cysts”, SMCs lack an epithelial lining and are better classified as diverticula containing cerebrospinal fluid (2). Three main types can be recognized based on criteria adapted from human medicine: type I, extradural diverticula without nerve root involvement; type II, extradural diverticula involving the nerve roots (Tarlov cysts); and type III, intradural diverticula also known as spinal arachnoid diverticula (2). Type I lesions include type Ia diverticula arising from arachnoid herniation through a dural defect and type Ib diverticula connected to the terminal dural sac by a pedicle. In dogs, type II and III SMCs are most frequently described, with type III often associated with congenital malformations in brachycephalic breeds such as Pugs and French Bulldogs (2, 5). To date, no type Ia SMCs have been reported in veterinary medicine. Although a suspected type Ib case has been previously described, its potential causal relationship with TCS had not been previously examined (1, 3).

Tethered cord syndrome arises due to abnormal caudal or dorsal positioning and/or restricted movement of the spinal cord and meninges, most often in association with congenital abnormalities of the developing nervous system. This syndrome may arise from structural impediments that restrict the physiological movement of the conus medullaris, including various forms of neural tube defects such as spina bifida with myelomeningocele and dermoid sinus type VI. It may also occur as a consequence of intrinsic microstructural alterations of the *filum terminale* (FT), in which a shortened, fatty, or inelastic FT exerts abnormal traction on the conus medullaris and associated nerve roots (4, 6, 7).

During normal development, differential growth of the vertebral column and spinal cord results in regression of the distal cord segments, producing the conus medullaris, cauda equina and the FT through retrogressive differentiation (8–10). The FT itself arises from neuroglial and meningeal remnants of the regressed spinal cord, while the surrounding dural sac undergoes progressive caudal tapering and termination (8, 11, 12). In humans, disturbances in these coordinated processes, alongside derangements in primary or secondary neurulation, gives rise to a spectrum of spinal dysraphic disorders that commonly result in TCS (8–10, 13). Similar developmental derangements occur in dogs given the close spatial and temporal relationship between the spinal cord, FT, meninges and vertebrae during embryogenesis (14). Such disruptions may contribute to congenital malformations of the meninges and other regional anatomical structures.

Arachnoid diverticula involving the FT have not been reported in veterinary medicine and are only rarely described in human literature (2, 3, 15, 16). Situated within the terminal portion of the vertebral canal, these diverticula may displace or compress adjacent neural structures, and associated dural or arachnoid adhesions or abnormal tension transmitted through the FT may further restrict conus medullaris mobility and contribute to a tethered cord effect (17). This report describes a previously unreported FT-associated extradural arachnoid diverticulum in a dog with associated TCS and outlines its neurological presentation, advanced imaging findings, surgical management, and outcome.

## Case description

A 2-year-old male intact French Bulldog was presented with a 3-month history of episodic aggression suspected to be secondary to lumbar pain identified by the referring veterinarian. Prior to referral, medical management with gabapentin (Gabapentin^®^, Crescent Pharma Ltd, UK; 20 mg/kg PO q8h) and meloxicam (Metacam^®^, Boehringer Ingelheim, UK; 0.1 mg/kg PO q24h) resulted in partial clinical improvement.

Physical and orthopedic examination revealed no abnormalities. On neurological examination, the dog demonstrated normal mentation. Postural and gait assessment revealed uneven weight distribution, with the dog favoring his thoracic limbs and alternating between a narrow and wide based stance in the pelvic limbs when standing. A short-strided stilted gait and intermittent pelvic limb tremors were observed. Postural reactions, namely paw repositioning and hopping, were mildly delayed in the pelvic limbs and normal in the thoracic limbs. Patellar reflexes were increased bilaterally while withdrawal reflexes were diminished. Muscle mass was reduced in the pelvic limbs, predominantly affecting proximal muscle groups, with normal tone. Discomfort was elicited on palpation of the lumbar vertebral column and pain perception was intact. Given these findings, the neurological lesion was localized to the L4–S3 spinal cord segments or cauda equina.

Hematology and biochemistry panels were within normal limits. Magnetic resonance imaging (MRI) of the middle to caudal thoracic, lumbar, sacral, and first caudal vertebral segments was performed under general anesthesia using a 1.5-T scanner (SIGNA HDe, GE Healthcare). The protocol included a dorsal short tau inversion recovery (STIR) sequence, sagittal and transverse T2-weighted (T2w) acquisitions, transverse T2w fluid-attenuated inversion recovery (FLAIR) and pre-contrast T1-weighted (T1w) sequences. A transverse T1w sequence was obtained following intravenous administration of a gadolinium-based contrast agent, Gadoterate Meglumine (Dotarem^®^, Guerbet, UK; 0.1 mmol/kg IV). Dynamic lumbosacral assessment was performed using a three-dimensional sagittal fast imaging employing steady-state acquisition (FIESTA) sequence acquired in neutral, flexed, and extended positions.

**Figure 1:**
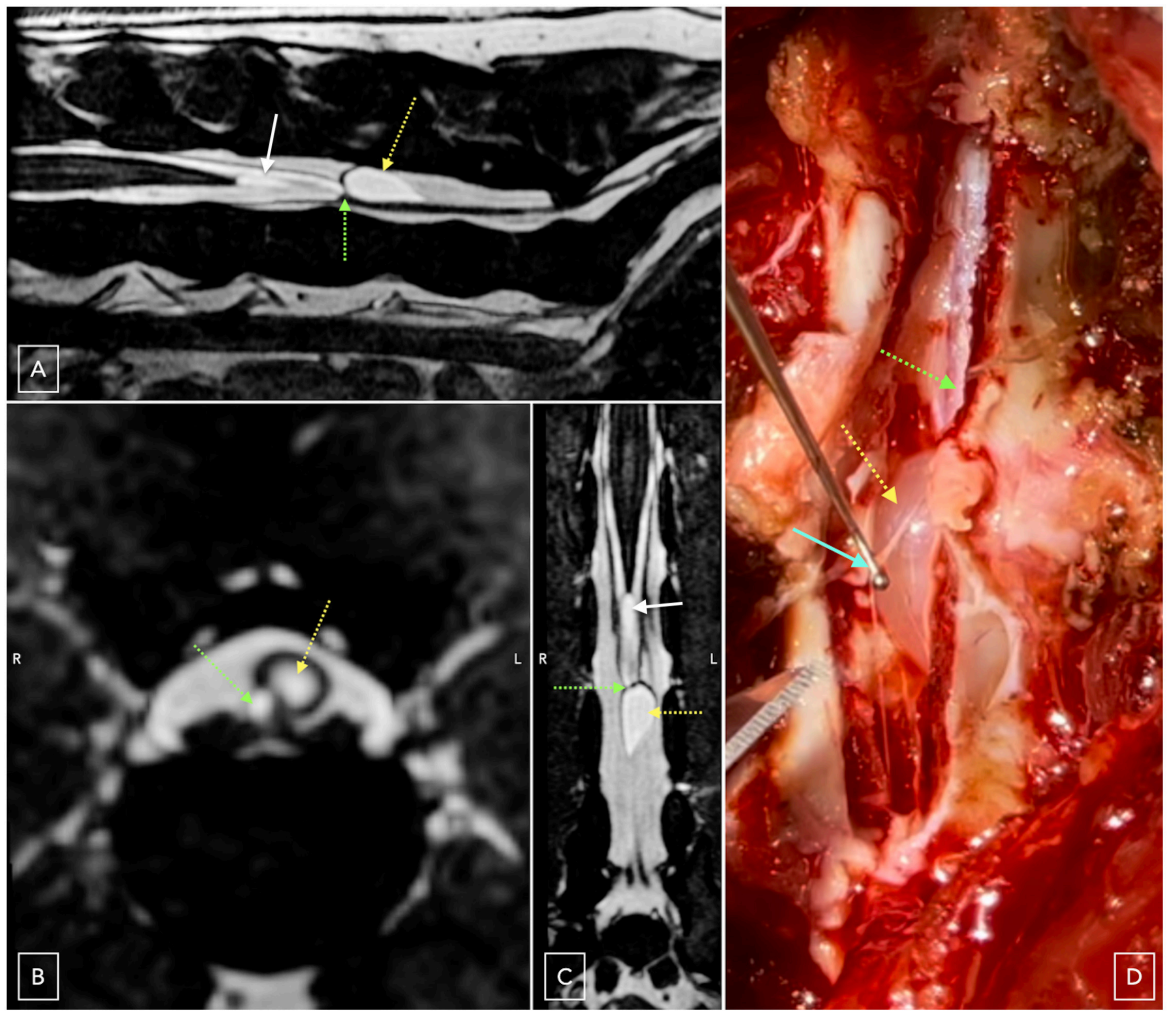
Three-dimensional reconstruction of a T2 weighted Fast Imaging Employing Steady-state Acquisition (FIESTA) sequence and corresponding intraoperative findings. The sagittal MRI plane **(A)** demonstrates a focal, well-defined, teardrop-shaped, CSF-isointense cyst-like extradural lesion at the level of L6 (yellow arrow), positioned dorsally within the vertebral canal and in direct contact with the termination of the dural sac (green arrow) and the origin of the filum terminale externum. An additional intradural CSF-isointense structure is visible in the region of the *filum terminale internum* within the subarachnoid space (white arrow). The transverse **(B)** and dorsal **(C)** reconstructed MRI planes further depict the dorsal location of the lesion (yellow arrows), its continuity with the meningeal structures, and its slight left-sided deviation relative to the dural sac termination (green arrows). On the dorsal reconstruction **(C)**, the intradural CSF-isointense structure appears elliptical and remains confined within the dural sac. Intraoperatively **(D)**, the cyst-like lesion (yellow arrow) is in direct apposition to the dural sac (green arrow) with a distinct fibrous dural-vertebral adhesion present along its dorsolateral aspect (cyan arrow), consistent with the imaging findings and supportive of restricted mobility of the caudal dural sac.

**Figure 2:**
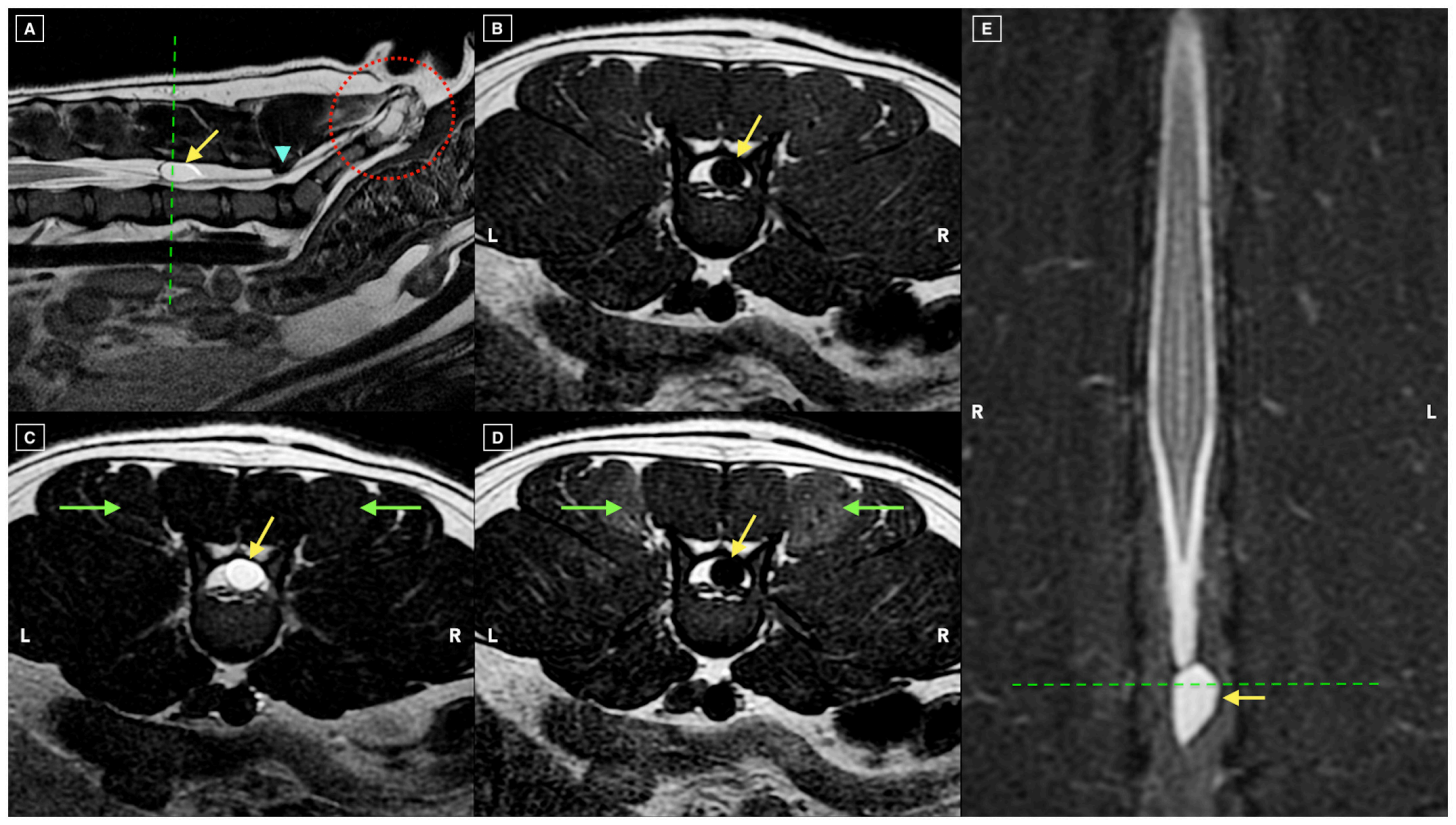
Multiplanar magnetic resonance images consisting of sagittal T2-weighted **(A)**, transverse T1-weighted **(B)**, transverse T2-weighted **(C)**, transverse post-contrast T1-weighted **(D)**, and dorsal STIR **(E)** sequences. A well-defined, teardrop-shaped, extradural cyst-like structure is present at the level of L6 (yellow arrows), positioned dorsally within the vertebral canal and in direct contact with the termination of the dural sac and the origin of the filum terminale externum, resulting in mild ventral displacement of the latter. The lesion is isointense to CSF, appearing hyperintense on T2-weighted **(A, C)** and STIR **(E)** sequences, hypointense on T1-weighted images **(B)**, and showing no contrast enhancement **(D)**, features consistent with a CSF-filled meningeal diverticulum. Additional findings include mild, symmetric enhancement of the dorsal sacrocaudal muscles compatible with denervation (green arrows, D), dorsal sacral laminar telescoping (blue arrowhead, **A**), a transitional S1 vertebra, and caudal vertebral dysgenesis (dotted red circle, **A**).

MRI identified a single, well-defined, extradural, teardrop-shaped structure measuring 1.4 cm in length, 0.7 cm in width, and 5.5 mm in height, extending from the cranial to the caudal aspect of L6. The lesion was positioned dorsally within the vertebral canal in direct contact with the dural sac termination and the origin of the FT *externum* (FTe), with slight left-sided deviation (Figures 1A–C). It resulted in mild ventral displacement of the FTe, which appeared subjectively thickened on sagittal images. The structure was isointense to CSF, showing T2w/STIR hyperintense and T1w/FLAIR hypointense signal, without contrast enhancement (Figure 2). Its cranial, broader portion occupied approximately 60% of the vertebral canal. The imaging characteristics were consistent with a meningeal extradural diverticulum or alternatively an extradural cystic lesion. Additionally, an intradural, well-demarcated, CSF-isointense lesion was identified within the subarachnoid space of the dural sac. The structure was positioned at mid-height within the dural sac, in the region of the FT internum (FTi) extending from the termination of the conus medullaris to approximately the mid-length of the FTi. It did not cause dilation of the dural sac and showed no contrast enhancement. On sagittal images, it appeared elongated to linear, while on transverse and dorsal reconstructions it was respectively rounded and elliptical. No compression of adjacent neural structures was observed (Figure 1). Differential diagnoses included focal subarachnoid compartmentalisation due to arachnoid septation or adhesion, a non-expansile intradural arachnoid diverticulum (SMC type III), or a filar cyst. A dilated ventriculus terminalis was considered unlikely given the apparent extramedullary location and absence of conus medullaris enlargement. Definitive classification could not be established based on MRI findings alone.

**Figure 3:**
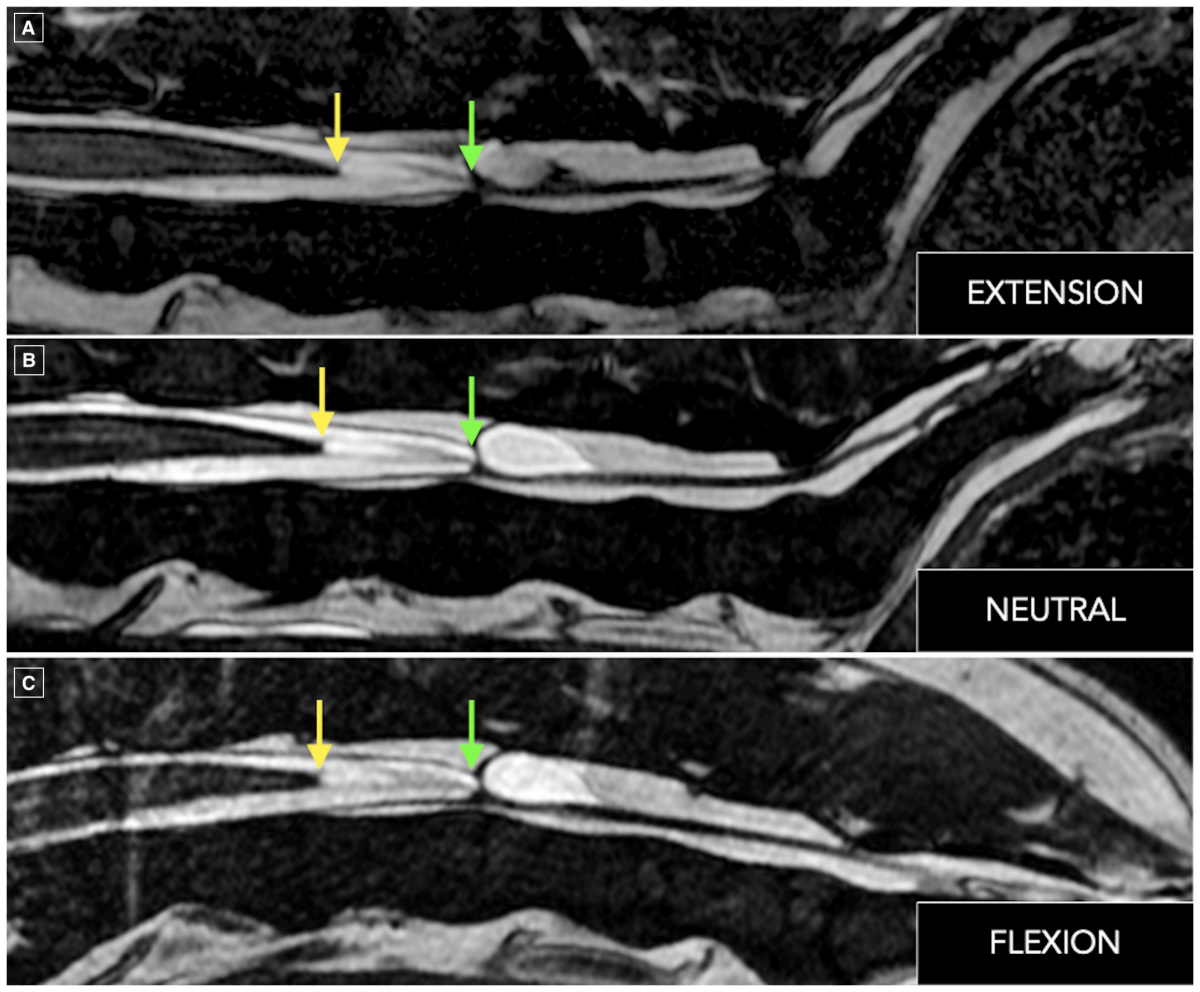
Dynamic lumbosacral magnetic resonance imaging using sagittal T2-weighted Fast Imaging Employing Steady-state Acquisition (FIESTA) sequences obtained in extension (lumbosacral angle = 135°; **A**), neutral positioning (lumbosacral angle = 141°; **B**), and flexion (lumbosacral angle = 170°; **C**). Across all positions, the termination of the conus medullaris (yellow arrows) remains at the cranial aspect of L5, while the termination of the dural sac (green arrows) remains at the L5–L6 intervertebral disc space, with no cranial or caudal translocation observed. The absence of positional mobility of these structures is consistent with restricted physiological motion and supports suspicion of tethered cord syndrome.

On dynamic lumbosacral MRI sequences, the conus medullaris terminated at the cranial aspect of L5, while the dural sac extended to the L5–L6 intervertebral disc space, positioned at approximately mid-vertebral canal height. Both the conus medullaris and dural sac terminations remained fixed in neutral, flexed, and extended lumbosacral angles, raising suspicion for TCS in association with the regional structural abnormalities identified (Figure 3).

Additionally, MRI demonstrated a transitional S1 vertebra and mild telescoping of its dorsal lamina accompanied by ventral bulging of the L7–S1 interarcuate ligament on neutral and extended views. Mild vertebral canal stenosis with effacement of the dorsal epidural fat in neutral and extended positions was visible, but not during flexion. No cauda equina compression was present in any of the acquired lumbosacral positions as per the available transverse images. The lateral dorsal sacrocaudal muscles showed bilateral symmetrical mild contrast enhancement, most consistent with denervation changes. The first lumbar vertebra had bilateral vestigial ribs, and the caudal thoracic vertebrae were shortened and irregular, consistent with hemivertebrae. Intervertebral discs showed diffuse T2w signal reduction consistent with degeneration, without spinal cord compression. A single, elongated, markedly dysplastic caudal vertebra was present caudal to the sacrum, showing an irregular contour, increased T2w hyperintensity and abnormal dorsal angulation toward the subcutaneous tissues, consistent with caudal vertebral dysgenesis, partial agenesis and congenital fusion of the first caudal segments.

The dog was re-admitted two days later for surgical management. A modified left-sided dorsolateral hemilaminectomy at L5–L6 was performed. At this level, a well-defined extradural meningeal cyst-like lesion was identified at the junction between the dural sac termination and the origin of the FTe. The lesion appeared to originate from a meningeal invagination at the junction of the dural sac termination and the origin of the FTe, without an identifiable dural breach, and was connected at this junction by a pedicle-like structure. A discrete fibrous strand of dural tissue was also noted along the left dorsolateral aspect of the dural sac and cyst-like lesion, forming an adhesion with the vertebral canal (Figure 1D). The cyst-like lesion was excised en bloc along the FTe and dural sac whilst preserving the integrity of the FTe. This required incising the dura of the FTe at its base using a No. 11 scalpel blade and extending the durotomy cranially over the dural sac termination. Resection of the dural adhesion was also performed. The FTi was identified within the dural sac following durotomy and appeared grossly unremarkable. Exploration of the intradural space did not reveal any additional abnormal structure corresponding to the previously described CSF-isointense intradural lesion, which may have decompressed or ruptured during dural opening. Cerebrospinal fluid flow appeared normal and no intradural adhesions were identified. The FTi was transected to ensure the restoration of physiological mobility of the conus medullaris in the event that excision of the cyst-like lesion alone proved insufficient.

A dorsal laminectomy at S1 vertebra was then performed and the interarcuate ligament at L7–S1 was removed, addressing the dynamic mild dorsal sacral laminar telescoping noted on imaging. A decellularized porcine small-intestine submucosa dural substitute (Biosis Healing Dural Repair Patch; Beijing Biosis Healing Biological Technology Co., Ltd., Beijing, China) was placed as a free on-lay graft over the dural defect without primary dural closure. The same graft was also applied over both laminectomy sites. Closure was routine, using polydioxanone (PDS II^®^, Ethicon, USA) 3/0 in a simple continuous pattern for the fascia, followed by poliglecaprone 25 (Monocryl^®^, Ethicon, USA) 3/0 in a simple continuous pattern for the subcutaneous tissues and the same material in an intradermal pattern for the skin. Post-operatively the dog received methadone (Comfortan^®^, Dechra, UK; 0.1 mg/kg IV q4h), ketamine (Anesketin^®^, Dechra, UK; 5 μg/kg/min CRI), paracetamol (Paracetamol Solution for Infusion^®^, B. Braun Medical, UK; 10 mg/kg IV q8h), gabapentin (20 mg/kg PO q8h), trazodone (Veenak, UK, 5 mg/kg PO q12h), and maropitant (Prevomax^®^, Dechra, UK; 1 mg/kg IV q24h). Ketamine was stopped over the following day, while methadone was continued for an additional day and then gradually weaned once the dog was comfortable.

**Figure 4:**
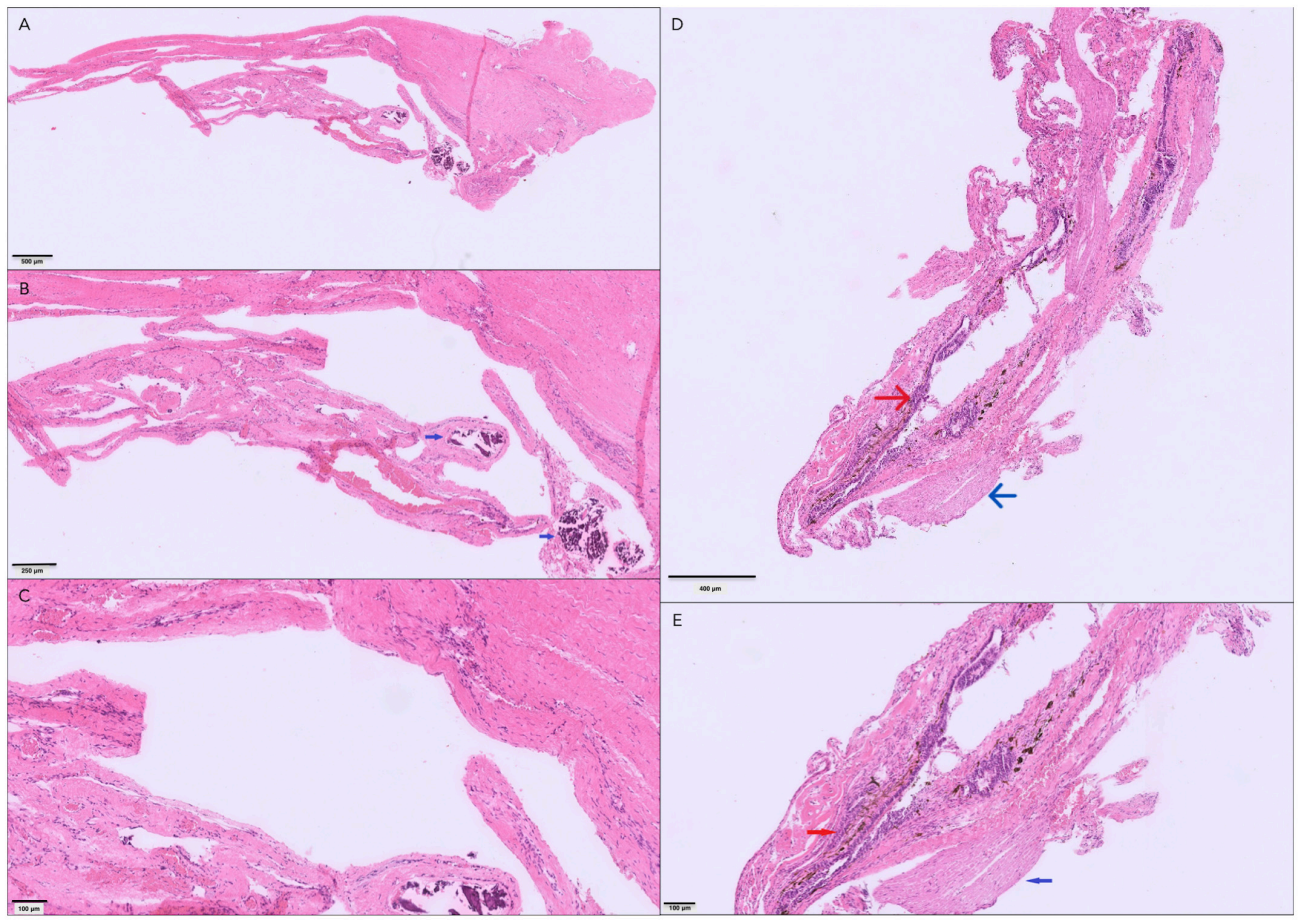
Histopathological sections of the cyst-like lesion connected to the meninges at the termination of the dural sac and to the filum terminale externum **(A, B, C)**, and of the filum terminale internum **(D, E)**, show: **(A)** dense collagenous connective tissue forming a broad outer layer consistent with the dura mater, overlying thinner, tortuous, and partially collapsed membranous layers interpreted as the arachnoidea; **(B)** multifocal, variably sized mineralised foci (blue arrows); **(C)** absence of an epithelial lining and lack of inflammatory infiltrates, findings that support interpretation as an arachnoid diverticulum. Panels **(D)** and **(E)** show a nerve fascicle (blue arrows) adjacent to dense collagenous connective tissue consistent with the filum terminale internum, containing a central lumen lined by well-differentiated ependymal cells (red arrows), compatible with the caudal continuation of the central canal of the spinal cord into the filum terminale.

Histopathological evaluation of the excised cyst-like lesion revealed a wall composed of dense collagenous connective tissue with distinct inner and outer meningeal layers and scattered small-caliber blood vessels, consistent with a diverticulum containing both dural and arachnoid elements (Figure 4). Additional sections included the fibrous strand of connective tissue identified intraoperatively that was compatible with dura mater. A separate sample contained tissue consistent with the FTi (Figure 4), characterized by nerve bundles and a centrally located ependymal-lined central canal, thereby confirming the identity of the structure transected during surgery (12). No inflammatory, degenerative, neoplastic, or infectious processes were identified.

The dog was discharged after 3 days of hospitalization on gabapentin (20 mg/kg PO q8h) and meloxicam (0.1 mg/kg PO q24h) with already improved pelvic limb posture, decreased stiffness and better withdrawal reflexes. Postoperative management included strict rest for the first 2–3 weeks, followed by continued avoidance of high-impact activity for 6 more weeks. Controlled low-impact exercise was permitted, and structured physiotherapy and hydrotherapy was recommended to support recovery. At 10 days postoperatively, the dog had notable clinical improvement, was comfortable and ambulating well, with significantly improved posture, gait, postural reactions, and pelvic limb withdrawal reflexes, and no pain on spinal palpation. At 1-year follow-up, the owner reported complete resolution of clinical signs, the dog remained pain-free and highly active, with no further episodes of aggression.

## Discussion

This case report describes the diagnosis and surgical management of an extradural arachnoid diverticulum consistent with a type Ib SMC associated with TCS in a French Bulldog. Both conditions are rare in dogs, and their simultaneous occurrence and possible mechanistic relationship has not previously been documented. This case therefore contributes valuable information on the recognition, diagnostic evaluation, and surgical management of this uncommon presentation involving the FT and associated meningeal structures.

The recognition and characterization of SMCs in dogs have advanced in recent years (1, 2). However, classification challenges persist, particularly in atypical presentations. Given the involvement of the FT in this case, initial differentials included a filar cyst or a variant of sacral terminal filar cyst, both described in human medicine. Filar cysts are transient, spontaneously regressive cystic dilatations at the cono-filar junction within the FT, most commonly identified in neonates and infants (15, 18). In contrast, sacral terminal filar cysts have been proposed as a subtype of type I SMC in which the FT lies within the cyst cavity (19). In the present case, a CSF-isointense intradural structure identified at the cono-filar junction initially raised consideration of a filar cyst; however, this finding was not confirmed intraoperatively and was not identified on histopathological examination of the FTi, precluding definitive classification as a filar cyst. Moreover, the extradural diverticulum arose as a focal outpouching of the meningeal coverings at the junction of the dural sac termination and the origin of the FTe and did not incorporate the FT within its walls, making these filar variants unlikely. Instead, the lesion is most consistent with a type Ib SMC, namely an extradural diverticulum arising from the caudal termination of the dural sac. Intraoperatively, it appeared pedunculated at this junction, a configuration supported by the preoperative imaging features and the histopathological findings.

Terminology is further complicated by the frequent conflation of type Ib meningeal diverticula with meningoceles (2). True meningoceles extend beyond the vertebral canal through an osseous defect and are associated with vertebral dysraphism, whereas type Ib diverticula are extradural arachnoid outpouchings confined within the vertebral canal, without associated spina bifida (1, 3, 14). The overlap in reported cases likely reflects shared congenital origins rather than identical morphology, consistent with the recognized predisposition of Bulldog-type breeds to congenital spinal anomalies, including meningoceles and spinal arachnoid diverticula (3, 5, 14).

A congenital origin for the lesions identified in this dog is supported by the developmental and spatial relationships among the terminal spinal cord, FT, meninges, and vertebrae. Although these structures arise from distinct embryological lineages, they develop within the same caudal region and during overlapping periods, making concurrent malformations possible (8, 12, 14). The distal spinal cord and FT form through primary and secondary neurulation, whereas the surrounding meningeal coverings and vertebrae differentiate from mesenchymal populations adjacent to the terminal neural tube. Disruptions in this caudal developmental region can therefore result in combined anomalies of the neuraxis, meninges, and vertebral column, as recognized in congenital conditions such as spina bifida with meningoceles in dogs (3). In the present case, the type Ib arachnoid diverticulum, the possible intradural subarachnoid lesion, the thickened FT, and the caudal vertebral dysgenesis are most plausibly interpreted as concurrent manifestations of such a regional congenital malformation complex within the spectrum of occult spinal dysraphism. Importantly, MRI demonstrated that the FTe terminated at the level of the vertebral dysgenesis, raising the possibility that the malformed vertebrae altered the normal distal anchoring of the filum. An abnormal distal attachment or altered alignment of the FTe could feasibly increase longitudinal tension along the filum, contribute to thickening, and restrict conus medullaris mobility, thereby producing a tethered cord effect (3, 14).

Pain associated aggression was a prominent presenting feature in this case and resolved completely following surgical intervention. Despite increasing recognition of pain-related behavioral change in veterinary medicine, its impact on animal welfare and the human-animal bond is still often underestimated (20, 21). Chronic pain in particular can profoundly impair a dog's quality of life; the development of allodynia, hyperalgesia and central sensitization can alter both cognitive and emotional processing, lowering the threshold for aggressive or defensive responses (21). Behavioral abnormalities such as anxiety, aggression, task avoidance, and self-mutilation have been associated with chronic pain secondary to spinal pathologies such as occult TCS and lumbosacral stenosis (6, 22). In the present case, early episodes of aggression were only provoked when the lumbar region was touched, but the behavior progressed to include reactions to handling other body regions and eventually unprompted aggression when in close proximity to the owner. This escalation reflects the chronic progressive nature of TCS and highlights how central sensitization can amplify and generalize pain perception beyond the site of pathology (6, 21). At 1 year follow up these aggressive episodes had entirely resolved. Early recognition of pain-related behavioral changes, coupled with timely investigation and treatment of the underlying condition is therefore essential in safeguarding patient welfare and preventing long term behavioral sequelae.

MRI was essential for characterizing the multiple abnormalities identified in this dog; however, several findings were considered incidental, as vertebral malformations such as hemivertebrae and caudal vertebral anomalies are highly prevalent in neurologically normal French Bulldogs (23, 24). In addition, the intradural lesion identified on MRI could not be confirmed intraoperatively nor on histopathological examination of the FTi, and its nature and clinical significance remain uncertain. Equally, the clinical relevance of the mild dorsal sacral laminar telescoping at L7–S1 was uncertain. Although this abnormality resulted in focal vertebral canal narrowing with dorsal epidural fat effacement, no definitive cauda equina compression was observed on transverse images, making a causal relationship with the presenting signs unlikely. Nevertheless, because dynamic effects could not be entirely excluded, an S1 dorsal laminectomy with resection of the interarcuate ligament was performed as a precautionary measure, recognizing that opportunities for repeat surgery in veterinary patients are limited.

Dynamic MRI provided additional diagnostic information in this case, as absent translocation of the conus medullaris and dural sac across positions raised suspicion for TCS as a potential complication of the FT extradural diverticulum. Absent conus medullaris translocation has also been reported in isolated canine case reports (25) and in a cohort of dogs with occult TCS (6), contrasting with studies demonstrating physiological cranio-caudal translocation of the conus medullaris in neurologically normal dogs (26). This possible relationship was not adequately explored in the single published case of a suspected type Ib SMC (1). Moreover, the FTe appeared subjectively thickened on MRI, a feature recognized in human TCS, where FT thickness > 2 mm or fatty infiltration is used as a diagnostic criterion (27). Despite these observations, dynamic MRI and filum thickness currently lack validated diagnostic criteria for TCS in dogs. No comparative studies have quantified conus or dural sac mobility in confirmed TCS vs. healthy controls, the normal range of physiological translocation remains unknown, and what constitutes a “fixed” conus or abnormally thickened filum is undefined. Interpretation is further complicated by difficulty in precisely identifying the conus–FT transition, particularly on high-resolution sequences where the FTi becomes more conspicuous, and by recognized breed variability in the level of conus termination (28). Moreover, the reliability of dynamic MRI, including intra- and interobserver agreement, has not been evaluated for this condition in dogs. Collectively, these limitations emphasize that dynamic MRI and FT assessment should be interpreted cautiously and only in conjunction with clinical history, neurological localization, and rigorous exclusion of alternative diagnoses. Future studies should establish normative reference ranges for conus and dural sac mobility, define FT morphometric criteria, assess observer reliability, and compare dynamic MRI and FT morphology findings between confirmed TCS cases and neurologically normal populations to determine diagnostic validity.

Surgical intervention was pursued to address the clinical signs and reduce the risk of further neurological deterioration (14). Effective management involved addressing both the extradural diverticulum and the associated TCS while minimizing manipulation of the surrounding spinal cord and nerve roots. The FTi was transected to maximize the likelihood of restoring physiological mobility of the terminal spinal cord. In the previously published case of a suspected type Ib SMC lesion, only the diverticulum was excised (1). Although pain and paraparesis resolved, fecal incontinence and pelvic limb ataxia persisted, with only partial improvement observed. Considering the FT was not transected, persistent tethering remains a plausible explanation for the residual deficits. In the present case, diverticulum excision combined with transection of the fibrous band as well as the FTi was followed by complete clinical recovery. Similar extradural adhesions are recognized in Bulldog-type breeds, and their resection has been associated with clinical improvement (14). Given the bundled nature of the intervention, causality cannot be assigned to any single surgical component, and it cannot be determined whether excision of the diverticulum and the fibrous band alone would have been sufficient to achieve the same outcome. In view of the limited feasibility of staged re-intervention in veterinary patients, all suspected contributors were addressed in a single procedure to maximize the likelihood of complete detethering. In human medicine, sectioning the FTi alone is typically sufficient to relieve TCS (29). In dogs, observational data suggest that cases in which both the FTi and FTe were sectioned appeared to have more favorable outcomes than those in which only the FTe was transected (6). On this basis, transection of the FTe was not performed in the present case. Overall, this case illustrates the importance of a tailored surgical approach that addresses all potential pathologies while acknowledging the limited evidence base that currently guides management of TCS in dogs.

An excellent clinical outcome was achieved following surgery, with resolution of pain and aggressive behavior. Postoperative complications such as persistent cerebrospinal fluid leakage and relapses secondary to retethering are well documented in humans (8, 30). Retethering has also been reported in dogs with occult TCS but was not observed in our case (6). At the 1-year follow-up, the dog remained in excellent health without neurological abnormalities; nevertheless, extended follow-up would be beneficial to monitor for potential recurrence. Although the temporal association between surgical intervention and clinical improvement supports a functional tethering mechanism, a direct causal relationship between the identified anatomical abnormalities and the observed clinical signs cannot be definitively established from a single case. Ideally, a postoperative dynamic MRI would help confirm restored craniocaudal mobility of the conus medullaris following sectioning of the FT, extradural diverticulum, and vertebral adhesion, but this was not pursued given the dog's stable condition and the anesthetic risks associated with repeat imaging.

## Conclusion

This case documents a previously unreported combination of an extradural arachnoid diverticulum (type Ib SMC) arising from the dural coverings at the junction of the FTe and the dural sac termination, together with associated TCS in a dog. It highlights both the diagnostic value and the current limitations of dynamic MRI in assessing conus medullaris mobility, emphasizing the need for cautious interpretation within the broader clinical and neurological context. Surgical management targeting all identifiable tethering structures resulted in an excellent clinical outcome, with complete resolution of pain and behavioral abnormalities and no recurrence at 1-year follow-up. Nonetheless, extended monitoring remains advisable, given the potential for retethering reported in both human and canine patients. Overall, this case underscores the importance of recognizing FT-associated meningeal diverticula as a possible cause of chronic lumbosacral discomfort in dogs and supports ongoing efforts to refine imaging criteria, optimize surgical approaches, and develop evidence-based guidelines for the diagnosis and management of TCS in veterinary practice.

## Data Availability

The original contributions presented in the study are included in the article/supplementary material, further inquiries can be directed to the corresponding author.

## References

[1] de Nies KS Edwards RA Bergknut N Beukers M Meij BP. Caudal lumbar spinal cysts in two French Bulldogs. Acta Vet Scand. (2018) 60:14. doi: 10.1186/s13028-018-0368-6 29490674 PMC5831591

[2] Lowrie ML Platt SR Garosi LS. Extramedullary spinal cysts in dogs. Vet Surg. (2014) 43:650–62. doi: 10.1111/j.1532-950X.2014.12200.x 24798122

[3] Da Costa RC Cook LB. Cystic abnormalities of the spinal cord and vertebral column. Vet Clin North Am Small Anim Pract. (2016) 46:277–93. doi: 10.1016/j.cvsm.2015.10.010 26706913

[4] Martín Muñiz L Del Magno S Gandini G Pisoni L Menchetti M Foglia A . Surgical outcomes of six bulldogs with spinal lumbosacral meningomyelocele or meningocele. Vet Surg. (2020) 49:200–6. doi: 10.1111/vsu.13342 31758707

[5] Mauler DA De Decker S De Risio L Volk HA Dennis R Gielen I . Signalment, clinical presentation, and diagnostic findings in 122 dogs with spinal arachnoid diverticula. J Vet Intern Med. (2014) 28:175–81. doi: 10.1111/jvim.12241 24428321 PMC4895525

[6] Espinosa Romero J De Decker S Santifort K Gutierrez-Quintana R Ortega M Uriarte A . Occult tethered cord syndrome: insights into clinical and MRI features, prognostic factors, and treatment outcomes in 30 dogs with confirmed or presumptive diagnosis. Front Vet Sci. (2025) 12:1588538. doi: 10.3389/fvets.2025.1588538 40717908 PMC12290461

[7] Yang C Lou X Huang L Ma Q Yin X Zhao Q . Accurate diagnosis and treatment of sacral meningeal cysts without spinal nerve root fibres: identifying leakage orificium using high-resolution spherical arbitrary-dimensional reconstructing magnetic resonance imaging. Front Neurol. (2024) 15:1298477. doi: 10.3389/fneur.2024.1298477 38356887 PMC10865724

[8] Hertzler DA DePowell JJ Stevenson CB Mangano FT. Tethered cord syndrome: a review of the literature from embryology to adult presentation. Neurosurg Foc. (2010) 29:E1. doi: 10.3171/2010.3.FOCUS1079 20593997

[9] Venkataramana NK. Spinal dysraphism. J Pediatr Neurosci. (2011) 6:S31–40. doi: 10.4103/1817-1745.85707 22069428 PMC3208922

[10] Acharya UV Pendharkar H Varma DR Pruthi N Varadarajan S. Spinal dysraphism illustrated; embryology revisited. Indian J Radiol Imaging (2017) 27:417–26. doi: 10.4103/ijri.IJRI_451_16 29379236 PMC5761168

[11] Jang HS Cho KH Chang H Jin ZW Rodriguez-Vazquez JF Murakami G. The filum terminale revisited: a histological study in human fetuses. Pediatr Neurosurg. (2016) 51:9–19. doi: 10.1159/000439284 26595116

[12] Posporis C Espinosa J Pumarola M Ortega EB Alomar J Santifort K . Anatomical and histological characterization of the filum terminale in dogs. Front Vet Sci. (2025) 12:1650893. doi: 10.3389/fvets.2025.1650893 40777825 PMC12330388

[13] deSouza RM Frim D Church P Elias T. Closed spinal dysraphism and tethered cord syndrome: A review of multidisciplinary team management. Adv Clin Neurosci Rehabil. (2014) 14:28–33. doi: 10.47795/GSGB7816

[14] Roynard P Dewey CW. Lumbosacral (myelo) meningoceles in dogs, related tethered cord syndrome, and their surgical management: review of the literature and clinical experience. Front Vet Sci. (2025) 12:1510800. doi: 10.3389/fvets.2025.1510800 40201081 PMC11975860

[15] Menezes AH Sato Y Dlouhy BJ Jones KA Moore SA. Ventriculus terminalis cyst in an infant: a case report. J Med Case Rep. (2023) 17:22. doi: 10.1186/s13256-023-03759-7 36683067 PMC9869499

[16] Lin G Yang C Yu T Zhang J Si Y Wu C . Sacral terminal filar cyst: a distinct variant of spinal meningeal cyst and midterm clinical outcome following combination resection surgery. Front Surg. (2023) 10:1272580. doi: 10.3389/fsurg.2023.1272580 38026491 PMC10654982

[17] Sun J-J Wang Z-Y Liu B Li Z-D Wu H-B Yen R-Y . Neck transfixion for sacral extradural spinal meningeal cysts without spinal nerve root fibers. Eur Spine J. (2016) 25:1945–52. doi: 10.1007/s00586-014-3471-z 25047654

[18] Irani N Goud AR Lowe LH. Isolated filar cyst on lumbar spine sonography in infants: a case-control study. Pediatr Radiol. (2006) 36:1283–8. doi: 10.1007/s00247-006-0317-9 17024492

[19] Li S Wu G Gu X Zhang D Zheng X. Surgical treatment of giant sacral terminal filar cysts: a case report and review of the literature. J Med Case Rep. (2025) 19:246. doi: 10.1186/s13256-025-05306-y 40405301 PMC12096574

[20] Mills DS Demontigny-Bédard I Gruen M Klinck MP McPeake KJ Barcelos AM . Pain and problem behavior in cats and dogs. Animals (2020) 10:318. doi: 10.3390/ani10020318 32085528 PMC7071134

[21] Malkani R Paramasivam S Wolfensohn S. How does chronic pain impact the lives of dogs: an investigation of factors that are associated with pain using the animal welfare assessment grid. Front Vet Sci. (2024) 11:1414314. doi: 10.3389/fvets.2024.1374858 38803796 PMC11129657

[22] Dodd T Jones J Holásková I Mukherjee M. Behavioral problems may be associated with multilevel lumbosacral stenosis in military working dogs. J Vet Behav. (2020) 35:8–13. doi: 10.1016/j.jveb.2019.07.010 32477020 PMC7259540

[23] Ryan R Gutierrez-Quintana R Ter Haar G De Decker S. Prevalence of thoracic vertebral malformations in French bulldogs, Pugs and English bulldogs with and without associated neurological deficits. Vet J. (2017) 221:25–9. doi: 10.1016/j.tvjl.2017.01.018 28283076

[24] De Decker S Packer RMA Cappello R Harcourt-Brown TR Rohdin C Gomes SA . Comparison of signalment and computed tomography findings in French Bulldogs, Pugs, and English Bulldogs with and without clinical signs associated with thoracic hemivertebra. J Vet Intern Med. (2019) 33:2151–9. doi: 10.1111/jvim.15556 31407402 PMC6766535

[25] De Decker S Watts V Neilson DM. Dynamic lumbosacral magnetic resonance imaging in a dog with tethered cord syndrome with a tight filum terminale. Front Vet Sci. (2017) 4:00134. doi: 10.3389/fvets.2017.00134 28868301 PMC5563312

[26] Nam J Kang K Kim K Choi J Choi M Yoon J. Translocation of the conus medullaris during dynamic lumbosacral magnetic resonance imaging in dogs. Am J Vet Res. (2021) 82:554–9. doi: 10.2460/ajvr.82.7.554 34166091

[27] Yundt KD Park TS Kaufman BA. Normal diameter of filum terminale in children: *in vivo* measurement. Pediatr Neurosurg. (1997) 27:257–9. doi: 10.1159/000121263 9620003

[28] Sparks CR Robertson I Olby NJ. Morphometric analysis of spinal cord termination in Cavalier King Charles Spaniels. J Vet Intern Med. (2019) 33:717–25. doi: 10.1111/jvim.15437 30758868 PMC6430917

[29] Patel M Vetter M Simonds E Schumacher M Laws T Iwanaga J . Mechanical relationship of filum terminale externum and filum terminale internum: is it possible to detether the spinal cord extradurally? Childs Nerv Syst. (2018) 34:1767–70. doi: 10.1007/s00381-018-3837-3 29797063

[30] Bhimani AD Selner AN Patel JB Hobbs JG Esfahani DR Behbahani M . Pediatric tethered cord release: an epidemiological and postoperative complication analysis. J Spine Surg. (2019) 5:337–50. doi: 10.21037/jss.2019.09.02 31663045 PMC6787363

